# Eighth Report to the Legislature of Massachusetts

**Published:** 1852-01

**Authors:** 


*Eighth Report to the Legislature of Massachusetts, relating to the Registry and Returns of Births, Marriages and Deaths, in the Commonwealth, from May* 1, 1848, *to January* 1, 1850. By Amasa Walker, *Secretary of the Commonwealth,* pp. 130.

The state of Massachusetts has obtained the proud position of being the foremost one in the Union in sanitary investigations. The present volume, which exhibits a large amount of valuable statistical information, is the eighth prepared under the auspices of the state. Although it is issued under the name of the Secretary of the Commonwealth, yet that functionary, in presenting it to the legislature, says that he found, on entering office, that his "predecessor had employed Dr. Josiah Curtis, of this city, (Boston,) to make out this report from the returns received by him, and under his supervision, it has been completed."

To Dr. Curtis, then, rather than to the Secretary of State, are we to look for the authorship of this report, and we are happy to say that it could not have been confided to more able hands. Dr. Curtis, who is yet young in his profession, greatly distinguished himself by his report on the sanitary condition of Massachusetts, made to the American Medical Association, as a member of the Committee on Public Hygiene, at a time when Dr. Wynne was the chairman of that committee, and Professors Yandell and Harrison, and Drs. Parrish and Gaillard were his colleagues.

In the present report, the tables are prepared with great care, and embrace a variety of useful information, and the conclusions drawn from them, are presented with great precision and force of language.

